# Sodium Chloride, Migraine and Salt Withdrawal: Controversy and Insights

**DOI:** 10.3390/medsci9040067

**Published:** 2021-10-30

**Authors:** Ronald B. Brown

**Affiliations:** School of Public Health Sciences, University of Waterloo, Waterloo, ON N2L3G1, Canada; r26brown@uwaterloo.ca

**Keywords:** sodium chloride, salt intake, migraine headache, withdrawal symptoms, edema, fluid retention, sodium retention, medication overuse headache

## Abstract

This paper examines evidence implicating migraine headache as a withdrawal symptom of excessive sodium chloride intake. Emerging research in food addiction posits that food and drug addictions share common features, such as withdrawal symptoms. Salt (sodium chloride) meets the criteria for the diagnosis of substance dependence, including withdrawal in which the substance is used to relieve withdrawal symptoms. The premonitory symptoms of migraine include food cravings for salty foods, which can alleviate migraine pain. Edema, possibly related to large amounts of salt consumed in binge eating, can cause approximately four pounds of retained fluid. This amount of fluid is similar to the fluid retained before the onset of migraine headache, which may be accompanied by polyuria. This paper proposes that inhibited withdrawal from highly processed food intake, rich in salt, mediates an association between increased sodium chloride intake and relief from migraine headache pain. The relief from withdrawal symptoms could also be a mediating factor that explains the controversial findings inversely associating dietary sodium intake with migraine history. Moreover, the withdrawal of retained sodium and edema related to the use of nonsteroidal anti-inflammatory drugs may elucidate a potential mechanism in medication overuse headache. Further research is needed to investigate the pain experienced from sodium chloride withdrawal in migraine headache.

## 1. Introduction

Like many drugs, salt (sodium chloride) has therapeutic, toxic, and addictive effects [1]. Sodium chloride meets criteria for the diagnosis of substance dependence, first listed in 1994 in *Diagnostic and Statistical Manual of Mental Disorders-IV* (DSM-IV), and combined with substance abuse in DSM-V in 2013 under substance use disorders [2]. DSM-V criteria for substance dependence include health problems, cravings, unsuccessful attempts to quit or control the substance, and withdrawal in which the substance is used to relieve withdrawal symptoms. Withdrawal headaches can be caused by substances such as caffeine, cannabis, opioids, and exogenous estrogen in oral contraceptives [2,3]. This paper examines the evidence implicating migraine headache as a withdrawal symptom of excessive sodium chloride intake, with the aim of contributing potential preventative strategies for migraine headache by reducing dietary salt intake.

A grounded theory method was used in this perspective paper to select and analyze articles from the research literature [4]. Starting with a clean slate by removing all assumptions, the grounded theory investigation added rigor and objectivity to the paper. Articles were selected by relevant keyword searches using PubMed, Google and Google Scholar, Scopus, and other resources from the University of Waterloo Library. Findings from the selected articles were compared and synthesized into themes. Themes were eventually formed into associative, causative, and mediating relationships until a cohesive novel theory on salt and migraine headache emerged, grounded in evidence from the research literature.

## 2. Migraine, Salt Intake, and Fluid Retention

Migraine is a leading disabling condition, second worldwide only to lower back pain [5], and migraine is the world leading cause of disability in people under 50 years old [6]. Comorbidities in persons with high migraine frequency and high headache pain intensity include hypertension, stroke, chronic kidney disease, and cardiovascular disease [7], which are conditions related to excessive sodium intake [8]. The headache phase in migraine is preceded by premonitory (prodrome) and aura phases, and is followed by a postdrome phase. Premonitory symptoms include fatigue, irritability, mood and activity changes, and food cravings, all of which may last throughout all four migraine phases [9]. Increased thirst is another premonitory symptom in migraine [10], and food cravings identified in patients with migraine include salty foods [11]. An earlier clinical study of 20 patients with a history of migraine described 3 patients with a craving for sweet or salty foods, “which when taken in time, alleviated the attack” [12], a finding that is consistent with withdrawal symptom relief. Conversely, “fasting-induced headache is more common in patients with migraine” [13].

Observing generalized edema, “which is known to occur before and during the early part of an attack of migraine,” researchers reported that female patients with migraine “often volunteer the information that their skirts become tight or their legs swell; while a number of subjects notice an increase in body weight” [14]. Fluid retention without a known medical condition, known as idiopathic edema, can amount to approximately four pounds of retained fluid, possibly related to large amounts of salt consumed in binge eating subsequent to strict dieting [15]. Of relevance, patients with migraine had more than five-times more disordered eating attitudes than a control group [16]; migraine is prevalent in over 75% of female patients with eating disorders [17], and migraine is almost twice as prevalent in patients with anorexia nervosa and bulimia nervosa compared to the general population of females [18]. Total body obesity and abdominal obesity are also associated with a higher prevalence of migraine [19].

An amount of retained fluid similar to that in idiopathic edema occurs before the onset of migraine headache, which may be accompanied by polyuria [20]. An early experiment testing the consumption of large amounts of water found that urinary sodium excretion was higher in patients with migraine compared to normal participants [21], suggesting higher concentrations of sodium in migraine patients. Additionally, sodium permeability through the blood–brain barrier and blood–cerebrospinal fluid barrier increases during migraine [22].

The results from a randomized clinical trial comparing a Western dietary pattern and the Dietary Approaches to Stop Hypertension (DASH) diet found that lower sodium intake was associated with 31% lower odds of headache compared to higher sodium intake, regardless of dietary pattern [23]. Another sodium-reduction intervention was associated with a 41% reduced risk of headache compared to a control group in a 36-month follow-up of the Trial of Nonpharmacologic Interventions in the Elderly (TONE) [24]. Moreover, the highest rate of adherence to a DASH diet (consisting of lower sodium intake levels in 266 women referred to a headache clinic) was associated with 46% reduced odds of severe migraine headache compared to the lowest rate of adherence to the diet [25].

## 3. Sodium Chloride and Controversial Migraine Relief

A scalp periarterial saline injection in patients was demonstrated to have high pain relief (analgesic) efficacy in migraine [26], providing supporting evidence that sodium chloride withdrawal symptoms in migraine headaches can be relieved by retained sodium chloride and fluid. The researchers suggested that the “prolonged compression of scalp arteries” accounted for saline efficacy, likely affecting pain receptors in “the periarterial nociceptive afferents.” Moreover, pain and inflammation is relieved by nonsteroidal anti-inflammatory drugs (NSAIDs) [27], and these substances can cause sodium retention and edema [28].

Similar to the sodium withdrawal related to dietary sodium chloride intake, the withdrawal of retained sodium and edema related to NSAID use can elucidate a potential mechanism in medication overuse headache (MOH), a secondary, withdrawal or rebound headache which is a condition that commonly progresses in people with chronic migraine pain [29]. MOH is frequently seen in neurology clinics, and patients using NSAIDs for at least 15 days a month and >3 consecutive months can be susceptible to MOH as a secondary headache caused by sodium withdrawal. More research is needed in this area.

The relief from withdrawal symptoms can also be a mediating factor that explains controversial findings inversely associating dietary sodium intake with migraine history [30,31]. Analyzing the data of 8819 adults in the 1999–2004 National Health and Nutrition Examination Survey (NHANES), Pogoda et al. found a 7% reduced odds of migraine history associated with increasing sodium dietary intake in men, and also in women with a lower body mass index (BMI) [30]. To avoid confounding from medication overuse headache, the researchers excluded respondents who reported analgesic medication use during the most recent month. However, the researchers did not appear to consider confounding due to the relief from withdrawal symptoms from increasing sodium intake, although the researchers cautioned against the use of sodium to treat migraine [30].

## 4. Highly Processed Food Withdrawal

The minimum daily amount of sodium required by the body is 500 mg; 1500 mg of sodium is an adequate daily intake amount; increased chronic disease risk is associated with an intake of more than 2300 mg of sodium; and the average American consumes 3400 mg of sodium a day [32]. “Processing a food often involves the use of added ingredients, including sodium-containing additives,” and highly processed and restaurant food provides more than 70% of an American’s total dietary salt intake [33]. The emerging research in food addiction posits that food and drug addiction share common features and similar neural mechanisms, and food additives have attracted attention as potential addictive triggers [34]. To investigate the withdrawal symptoms of highly processed food in humans, researchers have developed the Highly Processed Food Withdrawal Scale (ProWS), “adapted from self-report measures of drug withdrawal” [35].

“Migraine is the most common acute and recurrent headache syndrome in children,” and approximately 20% of migraine patients have their first attack at less than 5 years of age [36]. Highly processed foods are commonly consumed by children, and researchers developed the Highly Processed Food Withdrawal Scale for Children (ProWS-C), “a psychometrically sound tool for assessing parent-reported withdrawal symptoms in children” [37]. Future studies should examine migraine in children and adults potentially linked to highly processed food withdrawal, based on scores from the ProWS-C and ProWS, respectively. 

**Figure 1:**
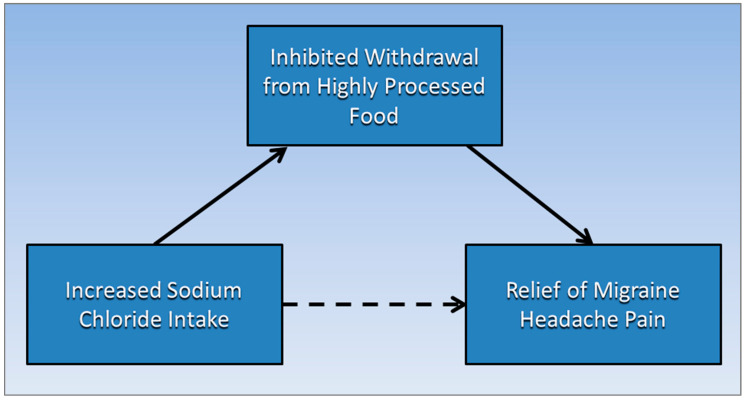
Inhibited withdrawal from highly processed food mediates the association of increased sodium chloride intake levels and the relief from migraine headache pain.

Figure 1 proposes that an association between increased sodium chloride intake levels and the relief from migraine headache pain (dotted arrow) is mediated by a causative pathway (solid arrows) running to and from inhibited withdrawal from highly processed food.

## 5. Conclusions

Sodium chloride has effects that meet the criteria for substance dependence, including withdrawal symptoms which are relieved by increased levels of sodium chloride intake. Migraine is associated with eating disorders, and Americans obtain most of their dietary sodium from highly processed foods which are high in sodium chloride additives. Although research studies confirm a positive link between migraine headache pain and sodium chloride intake, opposite findings are controversial. This paper proposes that the inhibited withdrawal from highly processed food mediates an association between increased sodium chloride intake levels and the relief from migraine headache pain. Sodium withdrawal related to NSAID use may also elucidate a mechanism in medication overuse headache. Further investigations are needed to clarify the role of sodium chloride dietary intake and withdrawal in the etiology and prevention of migraine.

## Data Availability

Not applicable.
