# Correction: Brown, R.B. Sodium Chloride, Migraine and Salt Withdrawal: Controversy and Insights. *Med. Sci.* 2021, *9*, 67

**DOI:** 10.3390/medsci10040066

**Published:** 2022-12-02

**Authors:** Ronald B. Brown

**Affiliations:** School of Public Health Sciences, University of Waterloo, Waterloo, ON N2L3G1, Canada; r26brown@uwaterloo.ca

## Text Correction

There was an error in the original publication [1]. Amount of sodium was incorrectly stated as sodium chloride.

A correction has been made to Section 4. Highly Processed Food Withdrawal, Paragraph Number 1:

The minimum daily amount of sodium required by the body is 500 mg; 1500 mg of sodium is an adequate daily intake amount; increased chronic disease risk is associated with an intake of more than 2300 mg of sodium; and the average American consumes 3400 mg of sodium a day [32]. 

The author states that the scientific conclusions are unaffected. This correction was approved by the Academic Editor. The original publication has also been updated.
